# Acute Respiratory Tract Infections (ARTIs) in Children after COVID-19-Related Social Distancing: An Epidemiological Study in a Single Center of Southern Italy

**DOI:** 10.3390/diagnostics14131341

**Published:** 2024-06-25

**Authors:** Raffaele Falsaperla, Vincenzo Sortino, Daria La Cognata, Chiara Barberi, Giovanni Corsello, Cristina Malaventura, Agnese Suppiej, Ausilia Desiree Collotta, Agata Polizzi, Patrizia Grassi, Martino Ruggieri

**Affiliations:** 1 Neonatal Intensive Care Unit and Neonatal Accompaniment Unit, San Marco Hospital, Azienda Ospedaliero-Universitaria Policlinico “Rodolico-San Marco”, University of Catania, 95121 Catania, Italy; 2 Unit of Clinical Paediatrics, San Marco Hospital, Azienda Ospedaliero-Universitaria Policlinico, “Rodolico-San Marco”, 95121 Catania, Italy; sortino.vinci@gmail.com (V.S.); ausilia.collotta92@gmail.com (A.D.C.); 3 Medical Sciences Department, University of Ferrara, 44124 Ferrara, Italy; mlvcst@unife.it (C.M.); sppgns@unife.it (A.S.); 4 Postgraduate Training Program in Pediatrics, Department of Clinical and Experimental Medicine, University of Catania, 95123 Catania, Italy; darialacognata@gmail.com; 5 Postgraduate Training Program in Pediatrics, University of Palermo, 90121 Palermo, Italy; chiara.barberi@hotmail.com; 6 Department of Health Promotion, Mother and Child Care, Internal Medicine and Medical Specialties “G. D’Alessandro”, University of Palermo, 90121 Palermo, Italy; giovanni.corsello@unipa.it; 7 Department of Educational Science, University of Catania, 95123 Catania, Italy; agata.polizzi1@unict.it; 8 Analysis Laboratory, San Marco Hospital, 95121 Catania, Italy; patricgrassi@gmail.com; 9 Unit of Clinical Pediatrics, AOU “Policlinico”, PO “G. Rodolico”, University of Catania, 95123 Catania, Italy; m.ruggieri@unict.it

**Keywords:** epidemiology, respiratory infections, BioFire^®^ FilmArray^®^ Respiratory Panel 2.1 Plus, multiplex PCR, RSV, rhinovirus, COVID-19, adenovirus, SARS-CoV-2, influenza A

## Abstract

In Sicily (Italy), respiratory syncytial virus (RSV), rhinovirus (HRV), and influenza virus triggered epidemics among children, resulting in an increase in acute respiratory tract infections (ARTIs). Our objective was to capture the epidemiology of respiratory infections in children, determining which pathogens were associated with respiratory infections following the lockdown and whether there were changes in the epidemiological landscape during the post-SARS-CoV-2 pandemic era. Materials and Methods: We analyzed multiplex respiratory viral PCR data (BioFire^®^ FilmArray^®^ Respiratory Panel 2.1 Plus) from 204 children presenting with respiratory symptoms and/or fever to our Unit of Pediatrics and Pediatric Emergency. Results: Viruses were predominantly responsible for ARTIs (99%), with RSV emerging as the most common agent involved in respiratory infections, followed by human rhinovirus/enterovirus and influenza A. RSV and rhinovirus were also the primary agents in coinfections. RSV predominated during winter months, while HRV/EV exhibited greater prevalence than RSV during the fall. Some viruses spread exclusively in coinfections (human coronavirus NL63, adenovirus, metapneumovirus, and parainfluenza viruses 1–3), while others primarily caused mono-infections (influenza A and B). SARS-CoV-2 was detected equally in both mono-infections (41%) and coinfections (59%). Conclusions: Our analysis underlines the predominance of RSV and the importance of implementing preventive strategies for RSV.

## 1. Introduction

Acute respiratory tract infections (ARTIs) represent a significant burden on infant health, often leading to illness and hospitalization. The clinical symptoms of respiratory infections frequently lack correlation with the causative pathogen [1]. While bacterial pathogens can cause ARTIs, the majority are viral [2]. Accurate differentiation between viral and bacterial etiologies is crucial for clinical management, including the judicious use of antibiotics [2,3,4], and predicting disease progression. Particularly, diagnosing lower respiratory tract infections such as pneumonia and bronchiolitis, which are major causes of morbidity and mortality in children worldwide, is imperative. Bronchiolitis, a common ARTI in young children [5,6,7], has long been associated with respiratory syncytial virus (RSV) as a primary respiratory pathogen [8,9]. Historically, distinguishing between viral and bacterial respiratory tract infections has been challenging as traditional methods such as culture, antigen detection, or serology are labor-intensive or lack sensitivity [10]. Polymerase Chain Reaction (PCR) methods, while more sensitive and specific, have not become the preferred diagnostic choice due to cost implications, particularly when targeting multiple agents [4]. Recently, multiplex assays like the BioFire^®^ FilmArray^®^ Respiratory Panel 2.1 Plus have emerged, offering rapid (∼60 min) detection of numerous pathogens directly from nasopharyngeal swab (NPS) samples [11]. The prevalence of common respiratory viruses—such as respiratory syncytial virus (RSV), parainfluenza virus (PIV), adenovirus (ADV), human metapneumovirus (MPV), human rhinovirus/enterovirus (HRV/EV), human bocavirus (HBoV), human coronavirus (HCoV), and influenza virus—has been extensively studied worldwide [4,12,13,14,15,16]. However, epidemiological data on ARTIs in Sicily and Italy, both before and after the SARS-CoV-2 pandemic, are limited. Our retrospective study aimed to evaluate the epidemiological patterns of ARTIs in children admitted to the pediatric emergency room or hospitalized for respiratory issues and/or fever at the Department of Clinical and Experimental Medicine, Pediatric Unit, San Marco Hospital, University of Catania in Sicily, Italy—a region in the South of Italy consisting of an island. Specifically, we sought to identify the predominant pathogens responsible for respiratory infections in pediatric patients in Southern Italy after the lockdown and assess whether the epidemiological landscape changed in the post-SARS-CoV-2 pandemic era. This investigation aimed to inform clinical practice with valuable epidemiological insights and contribute to the development of preventive measures, potentially including initiatives such as RSV vaccination or strategies to mitigate viral spread.

## 2. Materials and Methods

A retrospective single-center cohort study was conducted to assess the epidemiological trends in acute respiratory tract infections (ARTIs) at our tertiary referral pediatric center in Catania, Eastern Sicily, Italy. The study adhered to the Strengthening the Reporting of Observational Studies in Epidemiology (STROBE) statement, with all checklist items followed [17]. Upon admission to the hospital, a BioFire^®^ FilmArray^®^ test was performed using a nasopharyngeal swab to detect respiratory viruses, serving as the gold standard for diagnosing respiratory infections. Swab samples were collected from the oropharynx or nasopharynx using rotating swabs. Multiplex respiratory pathogen PCR data from children presenting with respiratory symptoms or fever to our Unit of Pediatrics and Pediatric Emergency in Catania, Sicily, were analyzed. This analysis aimed to capture the full seasonal dynamics of respiratory infections, which were exacerbated after COVID-19-related social distancing measures. 

The study period spanned fall–winter 2022/2023, from 1 September 2022 to 15 March 2023. All children who underwent a BioFire^®^ FilmArray^®^ Respiratory Panel 2.1 Plus test during this period were included in the analysis.

The analyzed cohort consisted of 204 subjects aged 1 month to 15 years. This retrospective study was approved by the local ethics committees (protocol code 32/2022, 25 March 2023). Fully informed consent was obtained from all the parents of the patients before recruitment.

The BioFire^®^ FilmArray^®^ Respiratory Panel 2.1 Plus was performed using a nasopharyngeal swab in each nostril and/or in oropharynx on all included patients; it diagnoses 19 types of viruses and 4 types of bacteria with a preparation time of approximately 2 min and a 60 min turnaround time. 

The following agents were analyzed:

− Influenza A (If A), A(H1), A(H3), A(H1)pdm09;
− Influenza B (If B);
− SARS-CoV-2 (SCOV2);
− MERS;
− Parainfluenza 1–4 (PIV1-4);
− Human metapneumovirus (MPV);
− Respiratory syncytial virus (RSV);
− Human rhinovirus (HRV)/enterovirus (EV)
  (the assay does not distinguish between these two pathogens);
− Adenovirus (ADV);
− Human coronavirus HCoV-HKU1;
− Human coronavirus HCoV-229E;
− Human coronavirus HCoV-OC43;
− Human coronavirus HCoV-NL63.

In addition, Mycoplasma pneumoniae, Chlamydia pneumoniae, Bordetella Pertussis, and Bordetella Parapertussis were included in the panel.

All statistical calculations were performed using Excel Ver. 2403 (build 17425.20176).

## 3. Endpoints

### 3.1. Primary Endpoint

The primary endpoint, also referred to as the true endpoint, aimed to identify the predominant respiratory pathogens responsible for infections in childhood.

### 3.2. Secondary Endpoints

As secondary endpoints, also termed surrogate endpoints, we investigated the following:

1. The incidence of respiratory infections in children.
2. The occurrence of coinfections and identification of the most common pathogen involved in coinfections.
3. Whether pathogens causing respiratory infections in children exhibited a higher incidence in mono-infections or coinfections.
4. Whether there were fluctuations in the peak incidence of viral infections throughout the examined months.

## 4. Inclusion and Exclusion Criteria

### 4.1. Inclusion Criteria

- Age range from >1 month to <15 years old.
- Presentation with acute fever (temperature ≥ 38 °C) or at least one respiratory symptom (such as rhinorrhea, nasal congestion, or sore throat);
- Undergoing the BioFire^®^ FilmArray^®^ Respiratory Panel 2.1 Plus test;
- Onset of illness within 3 days before hospitalization.

### 4.2. Exclusion Criteria

- Individuals with positive results from BioFire^®^ FilmArray^®^ Respiratory Panel 2.1 Plus tests conducted between 48 h after hospitalization and 3 days after discharge from our hospital, indicative of an infection contracted within the hospital rather than in the community.
- Patients hospitalized for another clinical condition.
- Patients with incomplete clinical information

## 5. Result

During the period from 1 September 2022 to 15 March 2023, a total of 204 BioFire^®^ FilmArray^®^ Respiratory Panel 2.1 Plus tests were collected from children aged 1 month to 15 years (mean age: 4.7 ± 3.9 years, 25th percentile: 2 months, 50th percentile: 1 year, 75th percentile: 4 years) at our Unit of Pediatrics and Pediatric Emergency in Catania, Sicily. Of these children, 52% were male and 48% were female, presenting with respiratory symptoms (cough and/or other symptoms suggestive of respiratory infections: rhinorrhea, nasal congestion, or sore throat) and/or fever.

### 5.1. Pathogens’ Prevalence in ARTIs: RSV Is the Most Frequent Virus in Childhood

**Figure 1:**
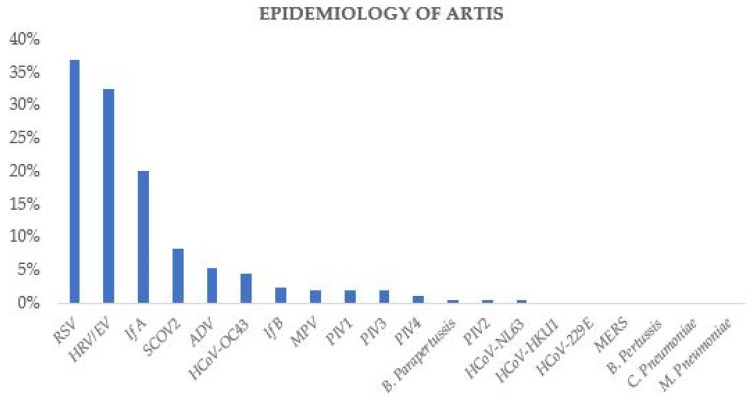
Epidemiology of acute respiratory tract infections during fall–winter 2022/2023 obtained from 204 Biofire^®^ FilmArray^®^ Respiratory Panel 2.1 Plus.

Out of the 204 swabs tested, 180 (88%) tested positive for one or more agents. The following agents were detected in order of frequency (*n*, % of single positives), as reported in Figure 1.

− RSV (*n* = 75, 37%);
− HRV/EV (*n* = 66, 32%)
− Influenza A (*n* = 41, 20%);
− SCOV2 (*n* = 17, 8.5%);
− Adenovirus (*n* = 11, 5.5%);
− Human coronavirus OC43 (*n* = 9, 4.5%);
− Influenza B (*n* = 5, 2.5%);
− Human metapneumovirus (*n* = 4, 2%);
− Parainfluenza virus 1 and 3, (*n* =4, 2%);
− Parainfluenza virus 4 (*n* = 2, 1%);
− Human coronavirus NL63 and parainfluenza virus 2 (*n* =1, 0.5%);
− Human coronavirus HKU1, 229E, and MERS were not detected.

During the 2022/2023 fall–winter period, only one specimen tested positive for Bordetella Parapertussis (*n* = 1, 0.5%), while B. Pertussis, Chlamydia Pneumoniae, and Mycoplasma Pneumoniae were not detected by the BioFire^®^ FilmArray^®^ throughout this period. Notably, viruses were predominantly responsible for acute respiratory tract infections (ARTIs), accounting for 99% of cases. Bordetella Parapertussis was the sole positive bacterial finding, identified in a single case. RSV (37%) was the most common agent found in our cohort, followed by human rhinovirus/enterovirus (32%), influenza A (20%), and SARS-CoV-2 (8.5%). Considering patients by age group, RSV was the most frequent virus in those ≤1 year of age, followed by HRV/EV, with influenza A in third place. In contrast, for patients > 1 year of age, HRV/EV was the most common virus, followed by influenza A, with RSV in third place.

### 5.2. Incidence of ARTIs in Children: Positive and Negative Rates of BioFire^®^ FilmArray^®^

**Figure 2:**
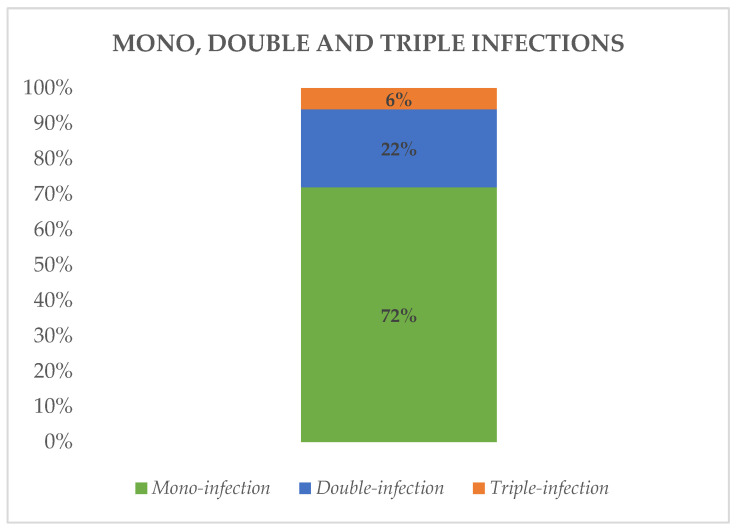
Percentage of mono-, double, and triple infections detected by Biofire^®^ FilmArray^®^ Respiratory Panel 2.1 plus.

Out of the 204 swabs analyzed, 180 (88%) tested positive for one or more agents. Of these, 130 samples (72%) contained a single agent, while 40 samples (22%) were positive for two agents, and only 10 samples (6%) were positive for three agents (Figure 2). A total of 24 children (12%) tested negative.

### 5.3. Occurrence of Coinfections and Principal Viruses Involved in Coinfections

Coinfection concerned 28% of total positive Biofire^®^ FilmArray^®^ Respiratory Panel 2.1 Plus. Among these coinfections, 80% were double infections and 20% were triple infections; no quadruple infections were detected.

**Figure 3:**
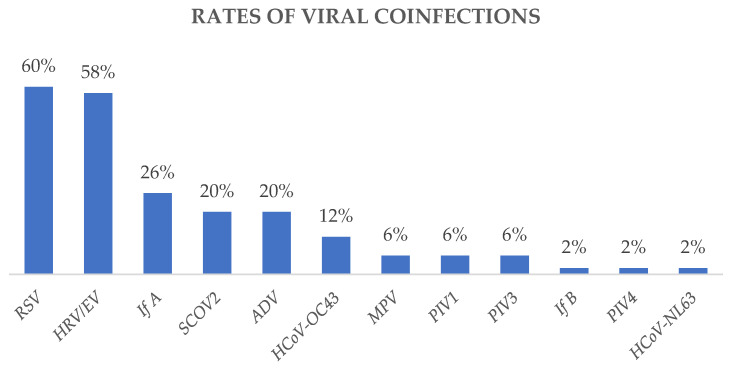
Rates of viruses involved in coinfections.

RSV and human rhinovirus/enterovirus were the predominant agents involved in coinfections. Among the 50 samples that tested positive for ≥1 viral agent, RSV was detected in 60% and human rhinovirus in 58%, followed by the other viruses (Figure 3).

### 5.4. Comparing Coinfection and Mono-Infection Rates for Each Virus Involved in ARTIs

**Figure 4:**
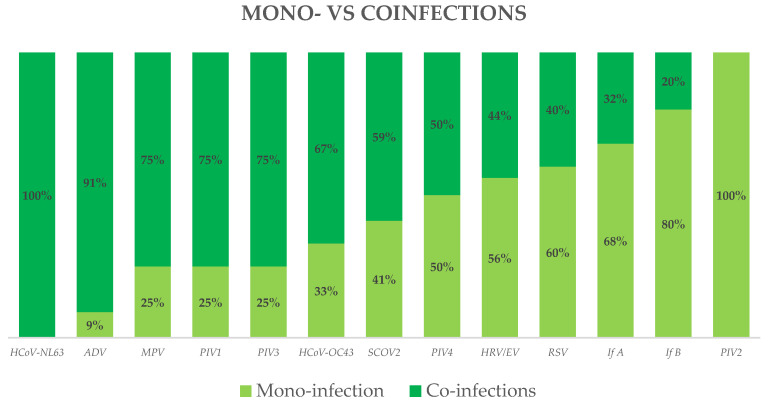
Distribution of viruses: mono-infection vs. coinfections.

**Figure 5:**
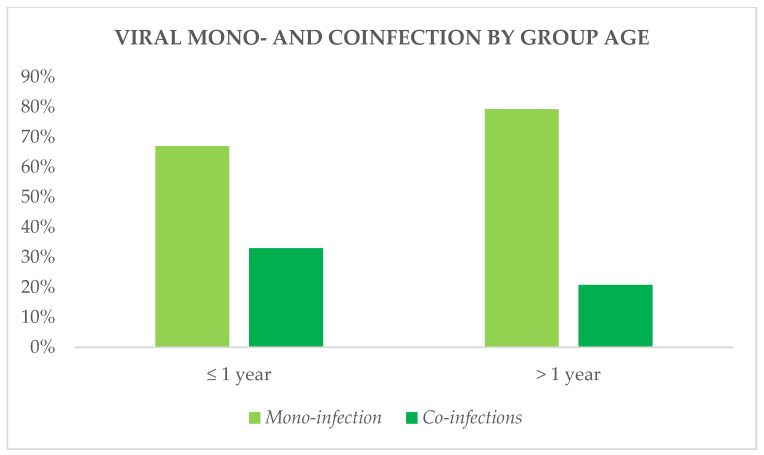
Mono-infection and coinfections by group age.

Human coronavirus NL63, adenovirus, metapneumovirus, and parainfluenza viruses 1–3 were detected almost exclusively in coinfections. SARS-CoV-2 was detected equally in both mono-infections (41%) and coinfections (59%), as well as parainfluenza virus 4 (50% vs. 50%) and human rhinovirus/enterovirus (60% vs. 40%). RSV, influenza A/B, and parainfluenza virus 2, furthermore, were identified at higher frequencies in mono-infections compared to coinfections (Figure 4). Coinfections were also identified more frequently in younger children (Figure 5).

### 5.5. Epidemiological Contrasts: Autumn (September–November 2022) vs. Winter (December 2022–March 2023)

Acute respiratory tract infections (ARTIs) experienced a notable increase, rising from 48 out of 58 (83%) positive samples in September–November 2022 to 130 out of 146 (89%) positive samples in December 2022 to March 2023. While RSV emerged as the predominant agent responsible for ARTIs throughout the entire fall–winter season of 2022/2023, it particularly dominated during the second trimester (December 2022 to March 2023), peaking in the first half of January 2023. Human rhinovirus/enterovirus exhibited greater prevalence than RSV during the first trimester of the season (September 2022 to November 2022). 

Except for RSV, the epidemic curves for all other viruses remained relatively stable throughout the entire epidemic season, excluding influenza B, metapneumovirus, SARS-CoV-2, and parainfluenza virus 1. Influenza B and metapneumovirus saw sudden emergence in December 2022 and January 2023, respectively, having not been detected earlier. Conversely, the rates of SARS-CoV-2 and parainfluenza virus 1 decreased during December 2022 to March 2023.

**Figure 6:**
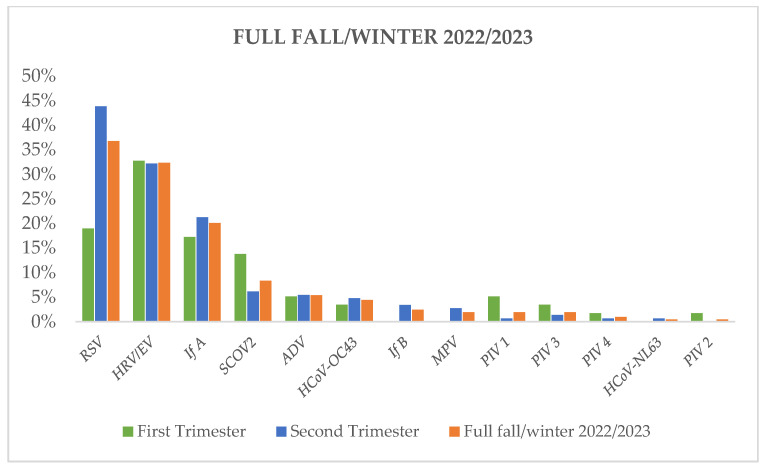
Distribution of viruses according to the timing of infection (first trimester vs. second trimester).

Influenza A, the most prevalent influenza virus of the season, similarly dominated as the third most detected virus throughout the epidemic season, maintaining a stable infection rate during both the first and second trimester (Figure 6).

## 6. Discussion

Viruses are the predominant pathogens involved in acute respiratory tract infections (ARTIs) [18], leading to severe morbidity, particularly among children [19]. Understanding the epidemiology of these viruses is crucial for efficiently managing and preventing these infections [20].

Our study depicted and subsequently analyzed the epidemiological data concerning respiratory viruses among children attending our Unit of Pediatrics and Pediatric Emergency in Catania, Sicily. The study period spanned the fall–winter of 2022–2023, marking the initial cold season following the relaxation of COVID-19-related social distancing measures in Italy.

During this timeframe, respiratory viruses such as influenza and respiratory syncytial virus, which had been significantly suppressed in Italy [21] in previous years due to measures such as hand hygiene and face masks, witnessed an unprecedented resurgence. This resurgence led to a notable uptick in viral infections and coinfections, not only within Italy but also in other countries [22].

In our cohort, respiratory syncytial virus (RSV) emerged as the most prevalent respiratory pathogen, with a positivity rate of 37%, consistent with findings from several other studies [23]. Following closely were rhinovirus at 32% and influenza A at 20%. While respiratory syncytial virus is globally recognized as a primary cause of hospitalization [24,25] and a significant contributor to child mortality [24,25], it does not always emerge as the most identified virus in respiratory infections among children [19,26,27,28]. Indeed, various epidemiological studies on pediatric patients have indicated that other viruses, particularly human rhinovirus/enterovirus, may be more commonly associated with children’s respiratory infections [19,26,27].

In Italy, there are currently limited epidemiological data available in the literature, with studies predominantly having been conducted before the onset of the COVID-19 pandemic. Specifically, the few epidemiological studies available were primarily conducted in Naples (Southern Italy), Rome (Central Italy), and Trieste (Northen Italy). Furthermore, there are no epidemiological data regarding viral infections in children in Sicily prior to the SARS-CoV-2 pandemic.

Regarding Naples, in a study [28] conducted in 2016 involving 356 pediatric patients, HRV/EV was identified in 44% of patients, followed by ADV at 18% and RSV at 13%. Another study [29] conducted after the COVID-19 pandemic, by contrast, showed a shift in epidemiological trends: RSV became the most detected pathogen (43.8%, with a peak observed in November 2021), followed by HRV/EV (25.9%) and PIV3 (10.8%).

A study [21] conducted in Rome revealed that throughout the entire study period (2018–2022), the most prevalent pathogen causing ARTIs was SARS-CoV-2 (38%), with rhinovirus and RSV ranking second and third, respectively. Specifically, the year 2020–2021 lacked a significant RSV peak in children, while in 2021–2022, RSV reached its highest observed peak during the study period.

A recent retrospective epidemiological study [30] conducted in Trieste showed that during the fall–winter of 2022–2023 (the same period as our study), rhinovirus was the most common agent, accounting for 40%, followed by RSV (30%) and influenza (25%) (Table S1, Supplementary File).

Although epidemiological studies in Italy are very limited in size, the literature (from Naples, Rome, and Trieste) consistently indicates an uptick in RSV incidence following the lockdown. However, it is worth noting that while in the post-lockdown phase, in the other parts of our country, RSV showed increased incidence but was never identified as the most frequent virus, in our study, similar to findings in Naples, RSV was indeed the most common virus of the season.

In terms of the global perspective, the literature presents considerable diversity, reflecting variations in the periods studied, the range of pathogens investigated, and the populations examined.

Wilson et al. [26] conducted a study (2014–2015) showing that HRV/EV was the most detected virus (RSV only 11%). 

Similarly, Khomenko et al. [31] reported analogous results in another study (2018–2020) conducted in Ukraine, with HRV/EV at 27.1% and ADV at 13.4% emerging as the most frequently identified viruses. Following these, RSV accounted for 13.2% and influenza A (If A) for 10.7%.

The prevalence of rhinovirus has been underscored in studies by Wilson and Khomenko conducted before the emergence of COVID-19. Recent evidence suggests that rhinovirus/enterovirus continues to be the most common pathogen responsible for respiratory infections in childhood, even in the post-COVID-19 era, in some parts of the world. A study by Weidemann et al. [22] (New York City, November 2021–December 2022) revealed that HRV/EV demonstrated the highest incidence throughout the investigated period, peaking in September 2022. Thus, it appears that the COVID-19 pandemic has not altered the most frequent pathogen responsible for ARTIs in children worldwide; this is in contrast with our findings.

However, the three most frequently identified viruses in children’s respiratory infections in the literature are HRV/EV, RSV, and If A [19,32,33]; this pattern was similarly affirmed in our study. 

In Italy, the epidemiology of respiratory viruses in children has undergone changes since the SARS-CoV-2 pandemic, marked by a rise in cases of RSV, which has not been documented elsewhere. In several countries worldwide, including Italy, the onset of the COVID-19 pandemic prompted the adoption of stringent social distancing measures, such as school closures and the promotion of hygienic practices. While designed to limit the spread of SARS-CoV-2, these measures also contributed to containing the transmission of other infectious agents, particularly respiratory viruses. 

In Italy, these social distancing measures were maintained for a longer duration compared to other parts of the world. Even after the extensive anti-SARS-CoV-2 vaccination campaign, the mandatory use of masks in public places persisted until June 2022 [34]. This prolonged adoption of social distancing measures could have been responsible for a deficit of immunization against various viral agents, triggering an epidemic resurgence of viruses such as RSV and influenza in subsequent periods. 

This immunological gap has been further exacerbated in Sicily, more than in the rest of Italy, due to the island’s geographical isolation. Sicily’s geographical exclusion from the rest of Italy has constrained social interactions and the transmission of respiratory pathogens, resulting in a great resurgence of RSV infections following the lockdown, as indicated by our study conducted during the fall–winter 2022–2023.

As for secondary endpoints, our retrospective study found that the incidence of respiratory infections in children is very high: a notable number of samples tested positive, with 180 out of the 204 swabs analyzed (88%) showing positive results for one or more agents. Children, due to their physical and immune vulnerability, are susceptible to rapidly transmitted and highly contagious viruses [27]; similar positivity rates, in fact, have been documented in other studies, indicating the widespread prevalence of respiratory viruses in pediatric populations. 

In our cohort, single infections were more prevalent than coinfections, accounting for 72% compared to 28%. Among cases of coinfections, 80% involved dual infections, while 20% involved triple infections. The pathogenic mechanisms of coinfections are often facilitated by a synergistic viral effect, and in some cases, coinfections may increase the risk of complications and severe clinical features [35,36]. It is noteworthy that coinfections are commonly observed in respiratory tract infections, especially in children [37,38]. This observation aligns with previous studies by Khomenko et al. [31] (79.5% for mono-infections and 20.5% for coinfections), Lei et al. [27] (82.0% single infections, 18.0% multiple infections), and He et al. [33] (31.1% coinfections). Similar findings were reported by Mandelia et al. [38], whose cohort showed a coinfection rate in the pediatric population of 35.0% (2068/5906), compared with only 5.8% (270/4591) in adults (Figure S1, Supplementary File). This trend could be attributed to the comparatively lower efficacy of the immune system in children, leading to a slower clearance of viral loads and an increased likelihood of detecting multiple pathogens in the airways during ARTIs. 

Our observations indicate that certain viruses tend to infect children more frequently as single infections, while others are more commonly found in coinfections. Specifically, adenovirus exhibited the highest coinfection positivity rate in our study: among the 11 cases of ADV detected, 10 (91%) were involved in co-detection with other viruses. In contrast, influenza B showed a low coinfection positivity rate (20%) compared to mono-infections (80%). Additionally, viruses like human coronavirus NL63 and parainfluenza virus 2 were exclusively detected in coinfections and mono-infections, respectively; however, they were found in only one patient each, so this finding was not statistically significant. These findings align perfectly with other studies [19,39]. Adenovirus is renowned for its extended viral shedding due to the robustness and stability of the viral DNA genome. This prolonged presence of the virus enhances the likelihood of co-detecting adenovirus. In contrast, viruses with less stable genomes may be eliminated more swiftly from the body, diminishing the probability of simultaneous detection with other pathogens.

The higher prevalence of SARS-CoV-2 coinfections observed in our study compared to the existing literature, such as the study by Edderdouri et al. [19] and the review by Weidmann [22], suggests a potential difference in the epidemiological dynamics of SARS-CoV-2 infections in our study population. This variation could be influenced by factors such as regional differences in viral circulation and the timing of data collection relative to the pandemic’s progression. While our research specifically focused on children, the studies by Edderdouri [19] and Weidmann [22] included both adults and children in their investigation. This variation in study populations could have influenced the observed rates of coinfection involving SARS-CoV-2 (Figure S2, Supplementary File). Our findings are consistent with a recent systematic review and meta-analysis by Krumbein et al. [40]. Consequently, it can be inferred that SARS-CoV-2, for reasons not yet fully elucidated, often plays a significant role in coinfections among children, more so than in adults.

Finally, we observed a distinct peak of RSV during the second trimester (December 2022 to March 2023) of the analyzed season, particularly in the first half of January 2023. The conventional winter peak linked to RSV has been consistently supported by numerous studies [39,41,42,43]. However, in a smaller percentage of studies, it has been observed that RSV may exhibit a peak in the autumn [19]. This variance could be attributed to differing climatic conditions that may favor the endemic survival of this virus.

Our study also revealed a moderated positivity rate for both influenza A and B, with rates of 20% and 2.5%, respectively, in our cohort. According to the literature [44,45,46], If A and If B had nearly disappeared or significantly declined in the two previous years, likely due to the implementation of social distancing measures aimed at mitigating the spread of COVID-19.

In our study group, influenza A consistently exhibited a sustained positive trend among children throughout the entire cold season. In contrast, influenza B remained absent until December 2022, when it abruptly surfaced.

## 7. Limits and Strength of the Study

Our study has certain limitations:

- The assay used in our study (BioFire^®^ FilmArray^®^ Respiratory Panel 2.1 Plus) does not distinguish between human rhinovirus and enterovirus and does not include the possibility of testing for EV-D68, a significant EV type.
- The sample size, although adequate for our center’s case studies, was relatively small, and the findings primarily pertain to our local population.
- Our study was performed in a small cohort of patients through a retrospective analysis.
- It was not possible to differentiate between superinfection and early coinfection in our study. Consequently, our observed coinfections may represent coinfection, sequential infection, contamination, or cross-reaction.
- Our study was conducted only in our center, and thus, the results are not generalizable to the country.
- On the contrary, our study boasts several strengths:
- Our study underscores the significance of employing BioFire^®^ FilmArray^®^ Respiratory Panel 2.1 Plus in both clinical practice and clinical trials. Notably, our case studies utilized a comprehensive respiratory panel featuring 23 pathogens, surpassing the scope of less recent studies that focused on only 7 viruses.
- We depicted the epidemiological progression of respiratory pathogens within our local context, offering insights that could potentially forecast future trends.
- We documented the viral epidemiological trends during the fall–winter 2022/2023 period in Italy where, notably, only a limited number of studies have undertaken such analyses, as many studies concluded their assessments up until the year 2022.
- Additionally, our findings indicate that, contrary to the prevailing literature, RSV surpasses rhinovirus as the primary pathogen responsible for ARTIs in our region among children, thus emphasizing the importance of RSV’s vaccination in our territory.
- Moreover, we observed that SARS-CoV-2 tends to contribute to coinfections rather than solitary infections in pediatric cases, deviating from the current literature.

## 8. Conclusions

In conclusion, our study endeavors to comprehensively elucidate the seasonal dynamics of respiratory infections in children during the fall–winter of 2022/2023 through the analysis of data derived from multiplex PCR tests utilizing the BioFire^®^ FilmArray^®^ Respiratory Panel 2.1 Plus. Our epidemiological investigation challenges conventional expectations, revealing a notable circulation of RSV in the Sicilian territory despite its milder climate. This underscores the imperative need to meticulously implement specific preventive strategies tailored for RSV in the Sicilian context. Further investigations with larger sample sizes are essential to enhance our understanding of viral epidemiological trends and the interplay of climatic and social factors. Our analysis underscores the importance of carefully implementing specific preventive strategies for RSV. In fact, contrary to expectations based on a milder climate, RSV shows significant circulation in the Sicilian territory.

## Data Availability

The datasets generated during and/or analyzed during the current study are available from the corresponding author upon reasonable request.

## Supplementary Materials

The following supporting information can be downloaded at: https://www.mdpi.com/article/10.3390/diagnostics14131341/s1; Table S1: The Strengthening the Reporting of Observational Studies in Epidemiology (STROBE) statement. Checklist of items that should be addressed in reports of observational studies [17]. Figure S1: Co-infections rates: Our data vs. literature. Figure S2: SARS-COV-2 in mono-infection and co-infection.
