# Supplementary material for: Acute Respiratory Tract Infections (ARTIs) in Children after COVID-19-Related Social Distancing: An Epidemiological Study in a Single Center of Southern Italy
Source: Diagnostics (Basel). 2024 Jun 25;14(13):1341. doi: 10.3390/diagnostics14131341 (PMC11240751; doi:10.3390/diagnostics14131341)
Supplement: Supplementary file 1 [file diagnostics-14-01341-s001.zip › diagnostics-2956695-supplementary.pdf]

The Strengthening the Reporting of Observational Studies in Epidemiology (STROBE) Statement
Checklist of Items That Should Be Addressed in Reports of Observational Studies

Item	N°	Recommendation	Page(s)	Notes
**Title and abstract**	1	(a) Indicate the study's design with a commonly used term in the title or the abstract.	1	This is a STROBE-conformed retrospective observational study of 204 children presenting with respiratory symptoms and/ or fever with age under 16 years of life that have performed BioFire® FilmArray® Respiratory Panel 2.1 Plus from 1 September 2022 to 15 March 2023
		(b) Provide in the abstract an informative and balanced summary of what was done and what was found.	1	Our objective was to capture the epidemiology of respiratory infections in children determining which pathogens were associated with respiratory infections following the lockdown, and whether there were changes in the epidemiological landscape during the post Sars-CoV-2 pandemic era. Materials and Methods: We analysed multiplex respiratory viral PCR data (BioFire® FilmArray® Respiratory Panel 2.1 PlusBioFire FilmArray® Respiratory Panel) from 204 children presenting with respiratory symptoms and/or fever to our Unit of Paediatrics and Paediatric Emergency. Results: Viruses were predominantly responsible for ARTIs (99%), with RSV emerging as the most common agent involved in respiratory infections, followed by Human Rhinovi-rus/Enterovirus and Influenza A. RSV and Rhinovirus were also the primary agents in co-

				infections. RSV predominated during winter months, while HRV/EV exhibited greater prev-alence than RSV during the fall. Some viruses spread exclusively in co-infections (Human Coronavirus NL63, Adenovirus, Metapneumovirus, and Parainfluenza viruses 1-3), while others primarily caused mono-infections (Influenza A and B). SARSars-CoVv-2 was detected equally in both mono-infections (41%) and co-infections (59%).
**Introduction**<br>Background/rationale	2	Explain the scientific background and rationale for the investigation being reported.	2	Acute respiratory tract infections (ARTIs) represent a significant burden on infant health, often leading to illness and hospitalization. Clinical symptoms of respiratory infections frequently lack correlation with the causative pathogen. While bacterial pathogens can cause ARTIs, the majority are viral. Accurate differentiation between viral and bacterial aetiologies is crucial for clinical management, including the judicious use of antibiotics, and predicting disease progression. Particularly, diagnosing lower respiratory tract infections such as pneumonia and bronchiolitis, which are major causes of morbidity and mortality in children worldwide, is imperative. Bronchiolitis, a common ARTI in young children, has long been associated with Respiratory Syncytial Virus (RSV) as a primary respiratory pathogen. Historically, distinguishing between viral and bacterial

				respiratory tract infections has been challenging, as traditional methods such as culture, antigen detection, or serology are labor-intensive or lack sensitivity. Polymerase Chain Reaction (PCR) methods, while more sensitive and specific, have not become the preferred diagnostic choice due to cost implications, particularly when targeting multiple agents. Recently, multiplex assays like the BioFire® FilmArray® Respiratory Panel 2.1 Plus have emerged, offering rapid (~60 minutes) detection of numerous pathogens directly from nasopharyngeal swab (NPS) samples.<br>The prevalence of common respiratory viruses—such as Respiratory Syncytial Virus (RSV), Parainfluenza virus (PIV), Adenovirus (AdV), human Metapneumovirus (hMPV), Rhinovirus (RV), human Bocavirus (hBoV), Human Coronavirus (HCoV), and Influenza —has been extensively studied worldwide. However, epidemiological data on ARTIs in Sicily and Italy, both before and after the SARS-CoV-2 pandemic, are limited.
Objectives	3	State specific objectives, including any prespecified hypotheses.	2	Our retrospective study aimed to evaluate the epidemiological patterns of ARTIs in children admitted to the Paediatric Emergency Room or hospitalized for respiratory issues and/or fever at the Department of Clinical and Experimental Medicine, Paediatric Unit, San Marco Hospital, University of Catania in Sicily, Italy—a region in

				the South of Italy consisting of an island. Specifically, we sought to identify the predominant pathogens responsible for respiratory infections in pediatric patients in Southern Italy after the lockdown and assess whether the epidemiological landscape changed in the post-SARS-CoV-2 pandemic era. This investigation aimed to inform clinical practice with valuable epidemiological insights and contribute to the development of preventive measures, potentially including initiatives such as RSV vaccination or strategies to mitigate viral spread
**Methods**				
Study design	4	Present key elements of study design early in the paper.	2-3	A retrospective single-center cohort study was conducted to assess the epidemiological trends of acute respiratory tract infections (ARTIs) at our tertiary referral pediatric center in Catania, eastern Sicily, Italy. The study adhered to the Strengthening the Reporting of Observational Studies in Epidemiology (STROBE) Statement, with all checklist items followed [17]. Upon admission to the hospital, a BioFire® FilmArray® test was performed using a nasopharyngeal swab to detect respiratory viruses, serving as the gold standard for diagnosing respiratory infections. Swab samples were collected from the oropharynx or nasopharynx using rotating swabs.

				Multiplex respiratory pathogen PCR data were analyzed from children presenting with respiratory symptoms or fever to our Unit of Pediatrics and Pediatric Emergency in Catania, Sicily. This analysis aimed to capture the full seasonal dynamics of respiratory infections, which were exacerbated after COVID-19-related social distancing measures. The study period spanned fall-winter 2022/2023, from September 1, 2022, to March 15, 2023. All children who underwent a BioFire® FilmArray® Respiratory Panel 2.1 Plus test during this period were included in the analysis.
Setting	5	Describe the setting, locations, and relevant dates, including periods of recruitment, exposure, follow-up, and data collection.	3	The study period was fall-winter 2022/2023 (spanned from 1 September 2022 to 15 March 2023) and all children who underwent a BioFire® FilmArray® Respiratory Panel 2.1 Plusl during this period were included.<br>The analysed cohort consists of 204 subjects aged 1 month to 15 years.<br>The BioFire® FilmArray® Respiratory Panel 2.1 was performed using a nasopharyngeal swab in each nostril and/or in oropharynx on all included patients;<br>The following agents were analysed:<br>- Influenza A (If A) (If A H1, If A H3, If A H1-2009),<br>- Influenza B (If B),

				- SARS-CoV-2 (SCOV2),<br>- MERS,<br>- Parainfluenza 1-4 (PIV1-4),<br>- Human Metapneumovirus (MPV),<br>- Respiratory Syncytial virus (RSV),<br>- Human Rhinovirus (HRV)/Enterovirus (EV)<br>(the assay does not distinguish between these two pathogens),<br>- Adenovirus (ADV),<br>- Human Coronaviruses HCoV-HKU1,<br>- Human Coronaviruses HCoV-229E,<br>- Human Coronaviruses HCoV-OC43,<br>- Human Coronaviruses HCOV-NL63.<br>In addition, Mycoplasma pneumoniae, Chlamydia pneumoniae, Bordetella Pertussis and Bordetella Parapertussis were included in the panel.
Participants	6	(a) Cohort study: Give the eligibility criteria, and the sources and methods of selection of participants. Describe methods of follow-up.<br>Case–control study: Give the eligibility criteria, and the sources and methods of case ascertainment and control selection. Give the rationale for the choice of cases and controls.<br>Cross-sectional study: Give the eligibility criteria, and the sources and methods of selection of participants.<br>(b) Cohort study: For matched studies, give matching criteria and number of exposed and unexposed.<br>Case–control study: For matched studies, give matching criteria and the number of controls per case.	4	Inclusion Criteria<br>• Age range from > 1 month to < 15 years old.<br>• Presentation with acute fever (temperature ≥ 38°C) or at least one respiratory symp-tom (such as rhinorrhoea, nasal congestion, or sore throat);<br>• Undergoing the BioFire® FilmArray® Respiratory Panel 2.1 Plus test;<br>• Onset of illness within 3 days before hospitalization.

				4.2. Exclusion Criteria:<br>• Individuals with positive results from BioFire ® FilmArray® Respiratory Panel 2.1 Plus tests conducted between 48 hours after hospitalization and 3 days after dis-charge from our hospital, indicative of an infection contracted within the hospital rather than in the community.<br>• Patients hospitalized for other clinical condition.<br>• Patients with incomplete clinical information<br>All patients were retrospective studied from clinical and laboratory point of view after they were admitted to the Pediatrics and Emergency Department according to the aim of the study.
Variables	7	Clearly define all outcomes, exposures, predictors, potential confounders, and effect modifiers. Give diagnostic criteria, if applicable.	4	The primary endpoint, also referred to as the true endpoint, aimed to identify the predominant respiratory pathogens responsible for infections in childhood.<br>As secondary endpoints, also termed surrogate endpoints, we investigated:<br><br>1.The incidence of respiratory infections in children.<br>2.The occurrence of co-infections and identification of the most common pathogen involved in co-infections.

				3.Whether pathogens causing respiratory infections in children exhibited a higher incidence in mono-infections or co-infections.<br>4.Whether there were fluctuations in the peak incidence of viral infections throughout the examined months.
Data sources/ measurement	8*	For each variable of interest, give sources of data and details of methods of assessment (measurement). Describe comparability of assessment methods if there is more than one group.	3-4	- sources of data: Medical records with laboratory value<br>- methods of assessment (measurement): BioFire® FilmArray® Respiratory Panel 2.1 Plus test
Bias	9	Describe any efforts to address potential sources of bias.	4	Considering the interference regarding the recent and remote pathological history reported by parents
Study size	10	Explain how the study size was arrived at.	4-9	Medical records were reviewed to identify eligible children according to the study project. In our database search, we found 204 Biofire® FilmArray® Repiratory Panel 2.1 Plus were collected from children aged from 1 month to 15 years.
Quantitative variables	11	Explain how quantitative variables were handled in the analyses. If applicable, describe which groupings were chosen, and why.	5	Biofire® FilmArray® Repiratory Panel 2.1 Plus were recorded with "positive" or negative" result. Results on Biofire® FilmArray® Repiratory Panel 2.1 Plus were recorded as a percentage.
Statistical methods	12	(a) Describe all statistical methods, including those used to control for confounding.<br>(b) Describe any methods used to examine subgroups and interactions.<br>(c) Explain how missing data were addressed.<br>(d) Cohort study: If applicable, explain how loss to follow-up was addressed.	4	All results were evaluated as the percentage ratio of each pathogen analysed to the total number of sample components.

		Case–control study: If applicable, explain how matching of cases and controls was addressed.<br>Cross-sectional study: If applicable, describe analytical methods taking account of sampling strategy.<br>(e) Describe any sensitivity analyses.		
**Results**				
Participants	13*	(a) Report the numbers of individuals at each stage of the study—e.g., numbers potentially eligible, examined for eligibility, confirmed eligible, included in the study, completing follow-up, and analyzed.<br>(b) Give reasons for nonparticipation at each stage.<br>(c) Consider use of a flow diagram.	4	In our database search, we found 204 patients according to our inclusion and exclusion criteria.
Descriptive data	14*	(a) Give characteristics of study participants (e.g., demographic, clinical, social) and information on exposures and potential confounders.<br>(b) Indicate the number of participants with missing data for each variable of interest.<br>(c) Cohort study: Summarize follow-up time—e.g., average and total amount.	4	Over the period 1 September 2022 to 15 March 2023, 204 Biofire® FilmArray® Repiratory Panel 2.1 Plus were collected from children aged from 1 month to 15 years (4.7 ± 3.9 SD years) (52% male and 48% female) presenting with respiratory symptoms (cough and/or other symptoms suggestive of respiratory infections: rhinorrhoea, nasal congestion, or sore throat) and/or with fever to our Unit of Paediatrics and Paediatric Emergency in Catania (Sicily).
Outcome data	15*	Cohort study: Report numbers of outcome events or summary measures over time.<br>Case–control study: Report numbers in each exposure category or summary measures of exposure.<br>Cross-sectional study: Report numbers of outcome events or summary measures.	4	The BioFire® FilmArray® Respiratory Panel 2.1 Plus performed using a nasopharyngeal swab in each nostril and/or in oropharynx on all included patients diagnoses 19 types of viruses and 4 types of bacteria:<br>- Influenza A (If A) (If A H1, If A H3, If A

				H1-2009),<br>- Influenza B (If B),<br>- SARS-CoV-2 (SCOV2),<br>- MERS,<br>- Parainfluenza 1-4 (PIV1-4),<br>- Human Metapneumovirus (MPV),<br>- Respiratory Syncytial virus (RSV),<br>- Human Rhinovirus (HRV)/Enterovirus (EV)<br>(the assay does not distinguish between these two pathogens),<br>- Adenovirus (ADV),<br>- Human Coronaviruses HCoV-HKU1,<br>- Human Coronaviruses HCoV-229E,<br>- Human Coronaviruses HCoV-OC43,<br>- Human Coronaviruses HCoV-NL63.<br>- Mycoplasma pneumoniae<br>- Chlamydia pneumoniae<br>- Bordetella Pertussis<br>- Bordetella Parapertussis
Main results	16	(a) Give unadjusted estimates and, if applicable, confounder-adjusted estimates and their precision (e.g., 95% confidence intervals). Make clear which confounders were adjusted for and why they were included.<br>(b) Report category boundaries when continuous variables were categorized.<br>(c) If relevant, consider translating estimates of relative risk into absolute risk for a meaningful time period.		Not applicable
Other analyses	17	Report other analyses done—e.g., analyses of subgroups and interactions and sensitivity analyses.	5-9	Out of the 204 swabs analysed, 180 (88%) resulted positive for one or more agents. The following agents were detected in order of

				frequency (n, % of single positives) as reported in figure 1:<br>- RSV (n =75, 37%),<br>- HRV / EV (n=66, 32%),<br>- Influenza A (n=41, 20%),<br>- SCOV2 (n=17, 8.5%),<br>- Adenovirus (n=11, 5.5%),<br>- Human Coronavirus OC43 (n =9, 4.5%),<br>- Influenza B (n=5, 2.5%),<br>- Human Metapneumovirus (n=4, 2%),<br>- Parainfluenza virus 1 e 3, (n =4, 2%),<br>- Parainfluenza virus 4 (n = 2, 1%),<br>- Human Coronavirus NL63 and Parainfluenza virus 2 (n =1, 0.5%),<br>- Human Coronaviruses HKU1, 229E and MERS were no detected.<br>-Only one specimen was positive for Bordetella Parapertussis (n =1, 0.5%) whereas B. Pertussis, Chlamydia Pneumoniae and Mycoplasma Pneumoniae were no detected by Biofire ® FilmArray® throughout the fall-winter 2022/2023 period.<br>Out of the 204 swabs analysed 180 (88%) resulted positive for one or more agents (Figure 2). Of these, 130 samples (72%) contained a single agent, while 40 samples (22%) were positive for two agents, and only 10 samples (6%) were positive for three agents (Figure 3). A total of 24 children (12%) tested negative.

				Co-infection concerned 28% of total positive Biofire ® FilmArray® Repiratory Panel 2.1 Plus: among these co-infections, 80% were double infections and 20% were triple infections; no quadru-ple-infection were detected (Figure 4). RSV and Human Rhinovirus/Enterovirus were the predominant agents involved in co-infections; among the 50 samples testing positive for ≥1 viral agent , RSV was detected in 60% and Human Rhinovirus in 58% following the other viruses (Figure 4).<br>Comparing Co-Infection and Mono-Infection Rates for Each Virus Involved in ARTIs: Human Coronavirus NL63, Adenovirus, Metapneumovirus, and Parainfluenza viruses 1-3 were detected almost exclusively in co-infections. SARSars-CoVv-2 was detected equally in both mono-infections (41%) and co-infections (59%), as well as Parainfluenza virus 4 (50% vs 50%) and Human Rhinovirus/Enterovirus (60% vs 40%). RSV, Influenza A/B and Parainfluenza virus 2, furthermore, have been identified at higher frequencies in co-infections compared to mono-infections (Figure 5).<br>Epidemiological Contrasts: Autumn (September-November 2022) vs. Winter (December 2022-March 2023):<br>Acute Respiratory Tract Infections (ARTIs) experienced a notable increase, rising from 48

				out of 58 (83%) positive samples in September-November 2022 to 130 out of 146 (89%) positive samples in December 2022 to March 2023.<br>While RSV emerged as the pre-dominant agent responsible for ARTIs throughout the entire fall/winter season of 2022/2023, it particularly dominated during the second trimester (December 2022 to March 2023), peaking in the first half of January 2023. Human Rhinovirus/Enterovirus exhibited greater prevalence than RSV during the first trimester of the season (September 2022 to November 2022).<br>Except for Human Rhinovirus and RSV, the epidemic curves for all other viruses remained relatively stable throughout the entire epidemic season, excluding Influenza B, Metapneumovirus, SARS-CoVv-2, and Parainfluenza virus 1. Influenza B and Metapneumovirus saw sudden emergence in December 2022 and January 2023, respectively, having not been detected earlier. On the other hand, the rates of SARS-CoV-2 and Parainfluenza virus 1 decreased during December 2022 to March 2023 (Figure 6)
**Discussion**				
Key results	18	Summarize key results with reference to study objectives.	7-15	The study period spanned the fall-winter of 2022-2023, marking the initial cold season following the relaxation of COVID-19-related

				social distancing measures in Italy.<br>Our retrospective study found a notable number of samples tested positive, with 180 out of the 204 swabs analysed (88%) showing positive results for one or more agents.<br>In our cohort, Respiratory Syncytial Virus (RSV) emerged as the most prevalent respiratory pathogen, with a positivity rate of 37%. Following closely were Rhinovirus at 32% and Influenza A at 20%.<br>Single infections were more prevalent than co-infections, accounting for 72% com-pared to 28%. Among cases of co-infections, 80% involved dual infections, while 20% in-volved triple infections; no instances of quadruple infections were identified in our cohort. RSV maintained its predominance not only in mono-infections (37%) but also in co-infections, where it constituted 60% of cases. Rhinovirus followed as the second most common pathogen in co-infections, comprising 58% of cases. Our observation revealed that Adenovirus, excluding Human Coronavirus NL63 (detected in 100% of cases in co-infection but found only in one case), was the virus with the highest co-infection positivity rate: of the 11 cases detected with ADVs in our study, 10 (91%) cases were involved in codetection. In contrast, Influenza B, excluding Parainfluenza virus 2 (detected in 100% of cases in mono-

				infection but found only in one case), had the lowest co-infection positivity rate (20%) compared to mono-infections (80%).<br>SARS-CoV-2 was identified more frequently in co-infections (59%) than in mono-infections (41%). This observation sharply contrasts with findings in the existing literature, where several studies report a low prevalence of SARS-CoV-2 in co-infections.<br>We observed a distinct peak of RSV during the second trimester (December 2022 to March 2023) of the analysed season, particularly in the first half of January 2023.<br>Our study revealed a moderated positivity rate for both Influenza A and B, with rates of 20% and 2.5%, respectively, in our cohort.<br>In our study group, Influenza A consistently exhibited a sustained positive trend among children throughout the entire cold season. In contrast, Influenza B remained absent until December 2022, when it abruptly surfaced. This occurrence might be attributed to the climatic conditions in Sicily.
Limitations	19	Discuss limitations of the study, taking into account sources of potential bias or imprecision. Discuss both direction and magnitude of any potential bias.	15	Our study has certain limitations:<br>-The sample size, although adequate for our centre's case studies, was relatively small, and the findings primarily pertain to our local population.<br>-Our study was performed in a small cohort of

				patients, through a retrospective analysis.<br>-It was not possible to differentiate between superinfection and early co-infection in our study. Consequently, our observed co-infections may represent coinfection, sequential infection, contamination, or cross-reaction.<br>-Our study was done only in our center and thus, the results are not generalizable to the country.
Interpretation	20	Give a cautious overall interpretation of results considering objectives, limitations, multiplicity of analyses, results from similar studies, and other relevant evidence.	13	Our study endeavours to comprehensively elucidate the seasonal dynamics of respiratory infections in children during the Fall/Winter of 2022/2023 through the analysis of data de-rived from multiplex PCR tests utilizing the BioFire® FilmArray® Respiratory Panel 2.1 Plus.
Generalizability	21	Discuss the generalizability (external validity) of the study results.	13	Our data suggest the importance of BioFire® FilmArray® Respiratory Panel 2.1 Plus in early detection of pathogen responsible for the disease to in order to correctly target the therapy and underlines the importance of carefully implementing specific preventive strategies for Respiratory Syncytial Virus (RSV). In fact, contrary to expectations based on a milder climate, RSV shows significant circulation in the Sicilian territory.
Funding	22	Give the source of funding and the role of the funders for the present study and, if applicable, for the original study on which the present article is based.		No funding was received.

*Give such information separately for cases and controls in case– control studies, and, if applicable, for exposed and unexposed groups in cohort and cross-sectional studies.

	**Catania, Sept22-March23**	**Trieste, Sept22-March23**[30]	**Napoli, July21-March22***[29]
**RSV**	37%	30%	43,80%
**HRV-EV**	32%	40%	25,90%
**If A**	20%	25%*	0,70%
**SCOV2**	8,50%	5%	2,20%
**ADV**	5,50%	7%	7,40%
**HCoV-OC43**	4,50%	not available	2,50%
**If B**	2,50%	25%*	0,10%
**MPV**	2%	not available	4,30%
**PIV1**	2%	4%*	not detected
**PIV3**	2%	4%*	10,80%
**PIV4**	1%	4%*	1,10%
**HCoV-NL63**	0,50%	not available	1%
**PIV2**	0,50%	4%*	0,10%
**HCoV-HKU1**	not detected	not available	not detected
**HCoV-229E**	not detected	not available	0,20%
**MERS**	not detected	not available	not detected
**B. Parapertussis**	0,50%	not available	not detected
**B. Pertussis**	not detected	not available	not detected
**C. Pneumoniae**	not detected	not available	not detected
**M. Pneumoniae**	not detected	not available	not detected
		*all Inf. viruses = 25%<br>*all Parainfl. viruses = 4%	***season 2022-2023 not available**

Table S1. EPIDEMIOLOGICAL TRENDS OF RESPIRATORY PATHOGENS IN CHILDREN IN THREE SINGLE CENTRES IN ITALY.

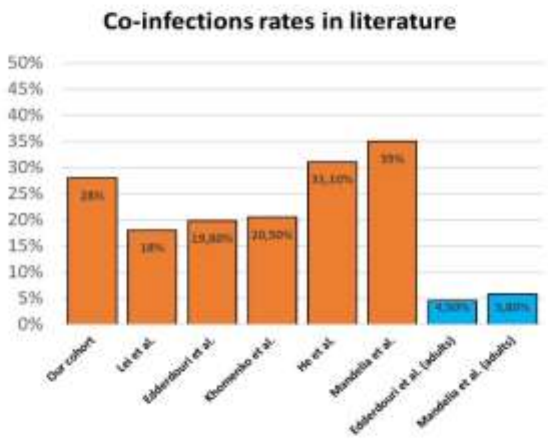

Figure S1. CO-INFECTIONS RATES: OUR DATA VS LITERATURE.

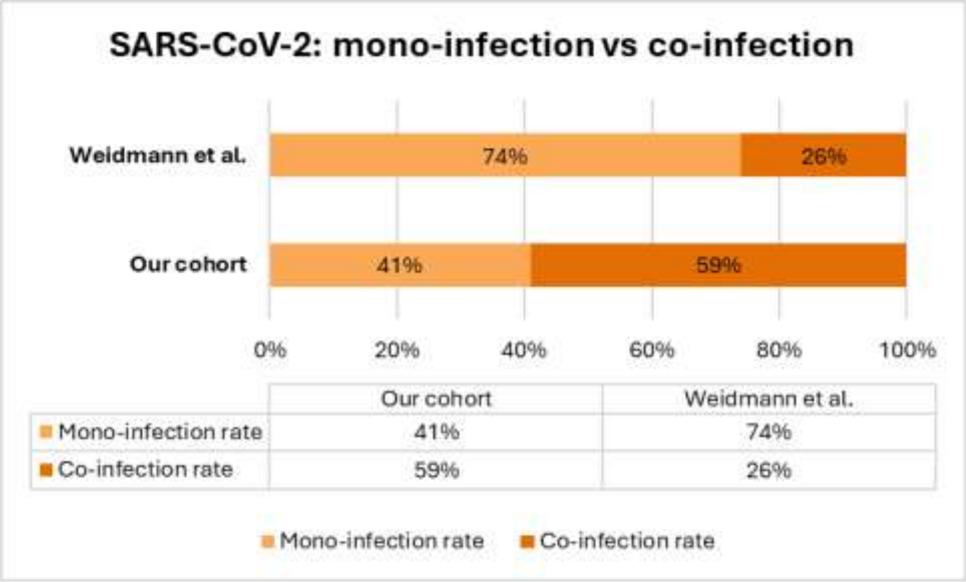

Figure S2. SARS-COV-2 IN MONO-INFECTION AND CO-INFECTION.
